# Enhancement of Surfactin yield by improving the medium composition and fermentation process

**DOI:** 10.1186/s13568-015-0145-0

**Published:** 2015-08-22

**Authors:** Judit Willenbacher, Wladimir Yeremchuk, Teresa Mohr, Christoph Syldatk, Rudolf Hausmann

**Affiliations:** Institute of Process Engineering in Life Sciences, Section II: Technical Biology, Karlsruhe Institute of Technology (KIT), Engler-Bunte-Ring 1, 76131 Karlsruhe, Germany; Institute of Food Science and Biotechnology (150), Section Bioprocess Engineering (150k), University of Hohenheim, Garbenstr. 25, 70599 Stuttgart, Germany

**Keywords:** Surfactin, Medium, Glucose, *Bacillus subtilis*, Fermentation, Fed-batch

## Abstract

Surfactin is one of the most promising biosurfactants due to its extraordinary surface activity. Commonly, the well-established Cooper medium, a glucose-based mineral salt medium, is utilized for the microbial production of Surfactin. The current study investigated the enhancement of Surfactin yields by analyzing the effects of different glucose concentrations, next to the introduction of an alternative chelating agent and nitrogen source. The utilization of 8 g/L glucose, 0.008 mM Na_3_citrate and 50 mM (NH_4_)_2_SO_4_ increased Surfactin yields from 0.7 to 1.1 g/L during shake flask experiments applying *Bacillus subtilis* DSM10^T^. Consequentially conducted shake flask experiments, employing five other Surfactin producer strains during cultivation in the former and enhanced version of the Cooper medium, suggest a general enhancement of Surfactin yields during application of the enhanced version of the Cooper medium. The enhancement of the medium composition is therefore most likely independent from the employed producer strain. The following utilization of the enhanced medium composition during fed-batch fermentation with integrated foam fractionation yielded 30 % more Surfactin in comparison to batch fermentations with integrated foam fractionation employing the former version of the Cooper medium.

**Electronic supplementary material:**

The online version of this article (doi:10.1186/s13568-015-0145-0) contains supplementary material, which is available to authorized users.

## Introduction

Research focusing on the microbial production of surfactants increasingly gains attention due to the strong surface activity and specific characteristics of some of these biosurfactants (Banat et al. 2010). Surfactin is one of the most highly discussed microbial surfactants as it exhibits a very low critical micelle concentration (0.036 g/L; Hirata et al. 2009) and lowers the surface tension of water to 27 mN/m (Arima et al. 1968). The lipopeptide consist of a peptide ring, comprising seven amino acids, and a β-hydroxy fatty acid. Many different characteristics of Surfactin are described, such as antitumor or antiviral activities (Kameda et al. 1974; Vollenbroich et al. 1997) but also induction of plants persistence against phytopathogens (Ongena and Jacques 2008) and insecticidal activity (Assié et al. 2002). Consequentially, there is a large number of possible applications of Surfactin e.g., in pharmaceuticals, agriculture, cosmetics, food industry or as a detergent.

Mostly the production of secondary metabolites in microorganisms is strongly influenced by the composition of the nutrients in their environment. Therefore, investigating a specific medium, with the aim to produce a certain molecule, usually becomes a highly discussed topic in its field of research. This also applies to the production of Surfactin with *B. subtilis*. Numerous suchlike studies, concerning the optimized medium or employing innovative carbon sources for the production of Surfactin, have been published (Peypoux et al. 1999). However, the typically utilized medium still demands further optimization as it does not comply with industrial standards, due to environmentally harmful components and substrate waste.

The shake flask experiments of Arima et al. (1968) using *B. subtilis* IAM 1213 were the benchmark for Surfactin production until the early 1980s (0.1 g/L). The Canadian group of Cooper et al. were the first to introduce an enhanced method for the production of Surfactin (Cooper et al. 1981), applying a foam fractionation process in a bioreactor but also introducing the first mineral salt medium for the production of Surfactin. The enhanced method led to a yield of 0.8 g/L Surfactin. Although some research groups used the semisynthetic “Landy medium” (20 g/L glucose, 0.1 % yeast extract; Nakano et al. 1988; Sandrin et al. 1990) the presented mineral salt medium, shortly after its introduction referred to as the “Cooper medium”, became the basis for most of the employed media to produce Surfactin until today (Horowitz et al. 1990; Qiu et al. 2014; Yakimov et al. 1995).

During the last 15 years, sustainable resources, especially for use in biotechnological processes, became more and more important. This is based on the aim to combine innovative, microbially produced products with sustainable industrial processes. From this perspective many research groups focused on alternative carbon sources for the production of Surfactin instead of using glucose, as suggested in the Cooper medium. Possible alternative substrates were rice straw and soybean flour, potato process effluent, cashew apple juice, rehydrated whey powder, cassava flour or peat hydrolysate (Cagri-Mehmetoglu et al. 2012; Davison et al. 2005; Freitas de Oliveira et al. 2013; Nitschke and Pastore 2004; Sheppard and Mulligan 1987; Zhu et al. 2013), all retaining low to moderate Surfactin yields (0.29–3.0 g/L). However, most studies did not further analyze the improvement of Surfactin production by calculating essential process values like substrate utilization (Y_X/S_, Y_P/S_) or specific (q_Surfactin_) and overall product yields (Y_P/X_). Other investigations focused on the productivity in respect of different sugars as carbon sources, proving glucose as the most effective (Abushady et al. 2005; Ghribi and Ellouze-Chaabouni 2011). On basis of these findings several studies investigated the optimal glucose concentration in mineral salt medium for the production of Surfactin or lichenysin with *B.* *subtilis* and *B.* *licheniformis*, respectively (Ghribi and Ellouze-Chaabouni 2011; Qiu et al. 2014; Sen 1997).

Sen (1997) analyzed the influence of glucose, NH_4_NO_3_, FeSO_4_ and MnSO_4_ in the Cooper medium on the production of Surfactin. The study was based on a 2^4^ full factorial central composite experimental design, allowing the analysis of four different parameters at the same time. The final result revealed 36.5 g/L glucose, 5 g/L NH_4_NO_3_, 4 × 10^−3^ g/L FeSO_4_ and 27.5 × 10^−2^ g/L MnSO_4_ as the optimized medium composition. In contrast, the study of Ghribi and Ellouze-Chaabouni (2011) investigated seven different glucose concentrations from 15 to 45 g/L in mineral salt medium and discovered 40 g/L glucose as the improved carbon source for Surfactin production with *B.* *subtilis* SPB1. The maximum yield was 0.72 g/L Surfactin. In contrast, a recent study (Qiu et al. 2014) investigated optimized glucose, NH_4_NO_3_ and buffer concentrations for the production of lichenysin. Five different glucose concentrations were analyzed from 10 to 50 g/L, where 30 g/L was identified as the improved glucose concentration.

Although several studies on enhanced glucose concentrations for the production of Surfactin have been conducted, there is no conclusive explanation why high glucose concentrations are required in mineral salt medium. In this sense further experiments were realized with the aim to optimize the Surfactin production. Further obstacles of the previously applied medium were the nitrogen source NH_4_Cl, which leads to an unnecessary accumulation of NaCl during pH control with NaOH, and the chelating agent EDTA, which is detrimental to the environment (Oviedo and Rodríguez 2003). As a consequence, experiments were conducted to analyze alternative substrates for the substitution of NH_4_Cl and EDTA. In summary, the aim of this study was to enhance the Surfactin yield by changing the medium composition and to prove a general production enhancement independent from the applied Surfactin producer strain by changing the medium composition.

## Materials and methods

### Microorganisms

The wild type strain *Bacillus subtilis* DSM 10^T^ was used in most experiments during the current study. A general enhancement of Surfactin productivity was tested employing the following *Bacillus* strains: DSM 3256, DSM 3258, DSM 1090, DSM 28227 and ATCC 21332. All microorganisms were obtained from the DSMZ (Deutsche Sammlung von Mikroorganismen und Zellkulturen GmbH, Braunschweig, Germany) or ATCC (American Type Culture Collection, Manassas, Virginia, USA).

### Culture conditions

#### Media

An overview about the different media used during this and previous studies is given in Table 1 (Cooper medium: A; slightly changed Cooper medium: B; enhanced medium: C). Stock solutions were used to assembly different medium combinations. The following final concentrations were adjusted every time: 0.03 M KH_2_PO_4_, 0.04 Na_2_HPO_4_, 0.0008 M MgSO_4_, 0.007 mM CaCl_2_, 0.004 mM FeSO_4_, 0.001 mM MnSO_4_. This implies that the buffer and trace element composition basically did not change throughout the experiments. In contrast, final concentrations of glucose varied widely (0, 2, 4, 6, 8, 10, 12, 15, 20, 30, 40, 50 g/L). Additionally, the substitution of NH_4_Cl (0.1 M; medium B) by (NH_4_)_2_SO_4_ (0.05 M; medium C) and the replacement of Na_2_EDTA (0.004 mM, medium B) with Na_3_citrate (0.008 mM, medium C) was performed to investigate a novel nitrogen source and chelating agent, respectively.

**Table 1:** Different media for the production of Surfactin with *Bacillus subtilis*

	Cooper medium	Modified after Cooper	Further optimized
	A	B	C
C	40 g/L glucose	40 g/L glucose	8 g/L glucose
N	50 mM NH_4_NO_3_	100 mM NH_4_Cl	50 mM (NH_4_)_2_SO_4_
Mg	0.8 mM MgSO_4_	0.8 mM MgSO_4_	0.8 mM MgSO_4_
Buffer	30 mM KH_2_PO_4_	30 mM KH_2_PO_4_	30 mM KH_2_PO_4_
	40 mM Na_2_HPO_4_	40 mM Na_2_HPO_4_	40 mM Na_2_HPO_4_
Trace elements	0.004 mM Na_2_EDTA	0.004 mM Na_2_EDTA	0.008 mM Na_3_citrate
	0.007 mM CaCl_2_	0.007 mM CaCl_2_	0.007 mM CaCl_2_
	0.004 mM FeSO_4_	0.004 mM FeSO_4_	0.004 mM FeSO_4_
	0.001 mM MnSO_4_	0.001 mM MnSO_4_	0.001 mM MnSO_4_

The original medium after Cooper et al. is shown in the first column (A). A slightly changed version of this medium was used throughout most experiments for previous studies (B). Hereby, the nitrogen source NH_4_NO_3_ was replaced by NH_4_Cl. During the current study the medium was further optimized to yield more Surfactin (C), employing less glucose (8 g/L), (NH_4_)_2_SO_4_ and Na_3_citrate

Fermentations were carried out with the final version of the optimized medium (Table 1: medium C). Stock solutions for the preparation of the bioreactor medium were prepared as described in Willenbacher et al. (2014), except for the concentration of the glucose stock solution which was about 48 g/L in 250 mL and the usage of 0.05 M (NH_4_)_2_SO_4_ and 0.008 mM Na_3_citrate. In contrast, to medium B (Table 1), medium C was limited by the amount of glucose. In order to cultivate for a similar amount of time, compared to cultivations in Willenbacher et al. (2014) (approximately 30 h), glucose was additionally fed to extend the cultivation time (from 20.83 h to 34 h). Therefore, a stock solution of 450 g/L glucose was prepared (23 mL) for inoculation after complete glucose consumption.

### Preparation of inoculum cultures

The precultures were incubated for 24 h at 30 °C and 120 rpm in a shake incubator chamber (Multitron II, HT Infors, Bottmingen, Switzerland). The 20 or 100 mL mineral salt medium for main culture (in 100 and 500 mL baffled shake flasks, respectively) were inoculated with a resulting OD_600_ between 0.05 and 0.1.

Two consecutive precultures were prepared for cultivations in benchtop bioreactors. The 500 mL baffled shake flasks, containing 100 mL of medium C, were inoculated to a resulting OD_600_ of 0.1. The benchtop bioreactors were inoculated from the second preculture to a resulting OD_600_ of 0.1.

### Shake flask cultivations

Cultivations in shake flasks were conducted to investigate different medium compositions and to analyze the Surfactin production of different *Bacillus* strains in medium B and medium C (Table 1). All shake flask experiments were performed as duplicates. In some cases the flasks were inoculated in a time-displaced way to collect samples of all cultivation phases. The cultivation duration varied between 30 and 50 h. Samples were taken by day every 2–3 h. The cultivation was stopped if the measured OD_600_ decreased after a significant growth phase.

### Cultivation in a 2.5 L benchtop bioreactor

Bioreactor cultivations were carried out as described in Willenbacher et al. (2014), using the same benchtop bioreactor system with pH, pO_2_ and temperature control (Minifors, HT Infors, Bottmingen, Switzerland). Since foam fractionation was applied, the foam was channeled through the exhaust cooler and collected in interchangeable bags. In contrast to Willenbacher et al. (2014), fermentations were conducted as fed-batch cultivations. After the depletion of glucose, the level of dissolved oxygen dramatically increased (because the cells suddenly experienced starvation). At this point a glucose stock solution was injected to increase the level of glucose in the bioreactor to the starting glucose concentration of approximately 8 g/L. As a result, the cultivation time was extended for 13.17 h. Samples were taken every 2 h accompanied by the exchange of the foam trap against a new collecting bag (applies only if foam was already leaving the bioreactor). All fermentations were performed as duplicates.

### Analytical methods

#### Sampling and sample processing

The samples taken during shake flask experiments were analyzed regarding OD_600_ (later on converted into cell dry weight (CDW) by division with the correlation factor 3; in case of *B. subtilis* DSM 3258 the pelleted growth prevented the determination of OD_600_ absorption) and Surfactin concentration (HPLC). The samples taken from the bioreactor were as well analyzed in respect of their OD_600_ (later indicated as CDW) and Surfactin concentration, but furthermore for their glucose concentration (enzymatic assay). Samples of the foam traps were also analyzed regarding their CDW, Surfactin and glucose concentration. The employed methods to quantify OD_600_, glucose, and Surfactin concentration were equivalent to the methods described in Willenbacher et al. (2014).

### Data analysis

An analysis of different process parameters allowed the evaluation of the applied fermentation process (fed-batch, Table 1: medium C) with earlier findings from cultivations in the original medium (batch, Table 1: medium B). Using the results of CDW, mass of glucose and mass of Surfactin, the values of Y_X/S_ [g/g], Y_P/X_ [g/g], Y_P/S_ [g/g], µ [h^−1^], q_Surfactin_ [g/(g h)], volumetric q_Surfactin_ [g/(L h)], Surfactin recovery [%], Surfactin enrichment and bacterial enrichment were determined as described in Willenbacher et al. (2014).

## Results

### Improvement of the copper medium to enhance Surfactin yields

The original Cooper medium (Cooper et al. 1981; Table 1: medium A) presents glucose as carbon source and NH_4_NO_3_ as nitrogen source. The buffer system is composed of KH_2_PO_4_ and Na_2_HPO_4_. Aside from that, the addition of MgSO_4_ serves as source for sulfur and magnesium. Additionally, the trace elements Fe, Ca and Mn are added together with the chelating agent EDTA. Early experiments contributing to previous studies (Willenbacher et al. 2014) were conducted with a slightly modified version of the Cooper medium (Table 1: medium B). The nitrogen source 50 mM NH_4_NO_3_ was exchanged against 1 M NH_4_Cl mainly because *Bacillus* prefers NH_4_ over NO_3_ as nitrogen source. Furthermore, the employment of NH_4_Cl solely required the analysis of one nitrogen compound. Additionally, the original 70 mM buffer system was replaced by a 10 mM buffer system when *B. subtilis* was cultivated in a benchtop bioreactor allowing pH control. In this way, it was possible to monitor the bacterial growth by the online acquisition of NaOH addition. The original glucose concentration was not altered, since earlier studies suggested 40 g/L as the optimal glucose concentration (Ghribi and Ellouze-Chaabouni 2011; Sen 1997).

Although repeatedly reliable results were obtained while employing the slightly changed Cooper medium (Table 1: medium B) this medium composition was further investigated to avoid unnecessary environmentally harmful components and substrate waste. As a consequence the chelating agent EDTA was replaced by citrate, which is a much more environmentally friendly and more favorable chelating agent. Furthermore, the nitrogen source NH_4_Cl was substituted by (NH_4_)_2_SO_4_, which prevents the accumulation of NaCl (caused by the addition of NaOH for pH control) inside the bioreactor and increases the amount of sulfur (which is comparably low in the original Cooper medium). Both substitutions did not affect bacterial growth or Surfactin productivity (data not shown).

The first shake flask cultivations revealed a higher Surfactin concentration at cultivations employing lower glucose concentrations. Subsequently, a shake flask experiment applying 0, 2, 4, 6, 8, 10, 12 and 15 g/L glucose was conducted encircling the improved glucose concentration for the production of Surfactin with *B. subtilis* DSM 10^T^ (the results of CDW and Surfactin yields are shown in the appendix, Additional file 1: Figure S1). The highest Surfactin yields were reached applying 6 and 8 g/L glucose (approximately 0.8 g/L Surfactin). These results surpassed Surfactin yields from shake flask cultivations employing the original 40 g/L glucose (0.6 g/L Surfactin).

**Fig. 1:**
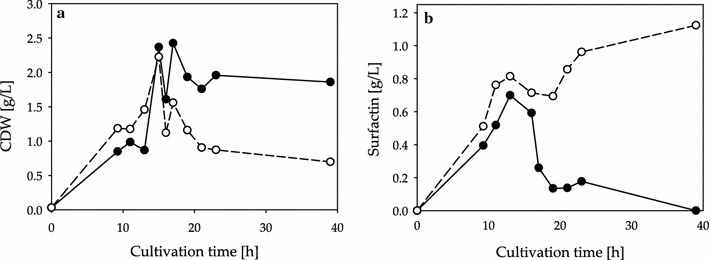
Time course of CDW and Surfactin concentrations of *B. subtilis* DSM 10^T^ during shake flask cultivations in medium B and further optimized medium C. The achieved CDW [g/L] is shown in **a**, whereas resulting Surfactin concentrations [g/L] are illustrated in **b**. The results of cultivation in medium B are given as *black dots*. Data from cultivations in medium C are presented as *white dots*. The cultivations were conducted as duplicates and in time-displaced flasks to illustrate a continuous course of growth and Surfactin production

Figure 1 compares the results of the former applied medium (slightly changed Cooper medium, Table 1: medium B) and the finally enhanced medium (Table 1: medium C). Time courses of the simultaneous cultivations of *B. subtilis* DSM 10^T^ in the two different media reveal a maximal CDW of 2.4 g/L in medium B and 2.2 g/L in medium C. Bacterial growth terminated after 15 h of cultivation in medium C as the further optimized medium is glucose limited. Nevertheless, a significantly higher concentration of Surfactin was produced in the further optimized medium C, yielding a maximum of 1.1 g/L Surfactin. In contrast, cultivations in medium B reached 0.7 g/L as maximal Surfactin concentration. This comparison shows a significant enhancement in Surfactin yields after medium optimization during shake flask cultivations.

### Does the optimized Cooper medium enhance Surfactin production in general?

To investigate whether the improved Surfactin yields during cultivation in the enhanced Cooper medium (Table 1: medium C) depend on the employed *Bacillus* strain DSM 10^T^, further shake flask experiments were conducted to analyze the Surfactin productivity of several other *Bacillus* strains in the optimized medium C.

The *B. subtilis* strains DSM 10^T^, DSM 28227, ATCC 21332, DSM 3256, DSM 1090 and DSM 3258 were analyzed regarding their Surfactin production during cultivation in medium B and medium C (Table 1). The CDW and Surfactin concentrations of these cultivations are shown in the appendix in Additional file 1: Figure S2. Most interestingly, the production of Surfactin seems to be enhanced in all cultivations employing the optimized medium C. Except for the results of DSM 3258, where the Surfactin production increased during cultivation in medium B. However, Surfactin production was generally very low (0.05–0.1 g/L) during the cultivations of DSM 3258 and the shown error bars indicate a rather similar production rate during cultivation in medium B and C. In summary, the concentration of Surfactin was doubled to tripled during cultivation of DSM 10^T^, DSM 28227, ATCC 21332, DSM 3256 and DSM 1090 employing the enhanced medium C in comparison to the previously applied medium B (sole exception DSM 3258), which suggests a general improvement of Surfactin production during cultivation in medium C independent from the applied *Bacillus* strain.

## Discussion

### Comparison with other studies analyzing the medium composition

As described earlier, Sen (1997) applied a design of experiment approach to analyze the influence of the medium components concentrations. In this fashion, solely three different concentrations were tested without altering the other three variables (NH_4_NO_3_, FeSO_4_ and MnSO_4_). In the case of glucose, three shake flask experiments were conducted with 0, 40 and 80 g/L glucose without changing the other medium components. This is a normal and common strategy when a design of experiment is approached, but covers a rather unrealistic range of glucose concentrations. Consequentially, shake flask cultivations containing no carbon source will not yield any Surfactin as cells are not able to grow properly. Furthermore, cultivations employing 80 g/L glucose should also be expected to yield low Surfactin concentrations as excess glucose concentrations negatively affect the growth behavior of *B. subtilis* (Dauner et al. 2001). The consequence is a much higher yield of Surfactin in cultivations with 40 g/L glucose. Consequently, 36.5 g/L was found to be the optimized glucose concentration.

Another important reference point is the applied method for the analysis of Surfactin yield. Sen (1997) determined the Surfactin yield via an indirect method measuring the surface tension. The relative Surfactin concentration was defined by serially diluting the culture broth until the critical micelle concentration (CMC) was reached. The number of dilutions which was necessary to start rising the surface tension was designated as CMC^−1^. Such indirect methods can be used to achieve a certain indication, but do not give specific information about the actual amount of product as the surface tension could be lowered by several other surfactants produced by *Bacillus* (e.g. Iturin or Fengycin).

The study of Ghribi and Ellouze-Chaabouni (2011) determined the Surfactin concentrations by approaching an indirect method as well. There, the precipitated and extracted crude product was weighed. This study identified 40 g/L as the most suitable glucose concentration as it yielded 0.72 g/L Surfactin. The determination of the Surfactin yield in this fashion is rather difficult, as shake flask experiments do not supply much product and during precipitation and extraction with chloroform and methanol lots of product is lost. Moreover, the precipitation with HCl (until reaching pH = 2.0) and following extraction with organic solvents does not necessarily lead to pure product.

In contrast, Qiu et al. (2014) applied HPLC to quantify the amount of produced lichenysin (1.25 g/L lichenysin with 30 g/L glucose in mineral salt medium). HPLC is the most accurate detection method, as the product is specifically identified by several peaks at characteristic retention times. Inevitable here is the application of a pure standard (e.g. Surfactin from Sigma-Aldrich). Qiu et al. (2014) were not able to purchase a lichenysin standard and therefore used Surfactin as a reference. Both lichenysin and Surfactin produce various isoforms, since different amino acids and fatty acids can be incorporated. It is therefore not very accurate to use Surfactin as HPLC standard for the detection of lichenysin. However, lichenysin was not commercially available at the time, hence Qiu et al. (2014) determined the lichenysin concentration as exactly as possible. Qiu et al. (2014) determined the product yield in the most accurate way in comparison to the other consulted studies. Nevertheless, the study identified 30 g/L glucose as the improved concentration for maximal product yield. The discrepancy between the results of the current study (8 g/L glucose as optimized concentration in the medium) and the study of Qiu et al. (2014; 30 g/L glucose) might be explained by the usage of two different strains (*Bacillus subtilis* and *Bacillus licheniformis*) and could also be referred to different regulation and expression of the *srfA* and *lchA* operons (the upstream region of the two operons seem to be similar but not identical, Sen 2010).

The results of Sen (1997), Ghribi and Ellouze-Chaabouni (2011) and Qiu et al. (2014) conflict the results of this study, which suggest 8 g/L glucose as enhanced concentration. The discrepancy could be explained by the fashion in which the experiments were conducted. All of the above studies focused either on glucose concentrations between 10 g/L and 50 g/L or used unsuitable low or high glucose concentrations (Sen, 1997). None of these studies incorporated experiments with 8 g/L glucose. Another reason could be the manner in which samples were taken. All of the discussed studies analyzed the Surfactin or lichenysin concentration at the end of cultivation. However, the product yield fluctuates greatly during the cultivation, which means maximal concentrations may have been missed. Additionally, detection methods (especially in the studies of Sen and Ghribi and Ellouze-Chaabouni) for the analysis of Surfactin lack specificity. Multiple applications of the optimized Cooper medium (Table 1: medium C) during cultivations of *B.* *subtilis* DSM 10^T^ and additional cultivations of further Surfactin producers in the current study proved a consistent enhancement of Surfactin yield. Therefore the following experiments were conducted in the improved medium C.

### Application of the optimized Cooper medium during cultivation in a 2.5 L benchtop bioreactor with integrated foam fractionation

The shake flask cultivations of *B. subtilis* DSM 10^T^ using the optimized medium (Table 1: medium C) reached significantly higher values for the production of Surfactin compared to results employing the former medium (Table 1: medium B). The results of the bioreactor cultivation of *B.* *subtilis* DSM 10^T^ applying foam fractionation in medium B (presented in Willenbacher et al. 2014) were already promising concerning Surfactin recovery, enrichment and total mass of Surfactin. After the final optimization of the medium another bioreactor cultivation of *B. subtilis* DSM 10^T^ with integrated foam fractionation was conducted to compare production rates of Surfactin with results obtained from cultivations presented in Willenbacher et al. (2014).

**Fig. 2:**
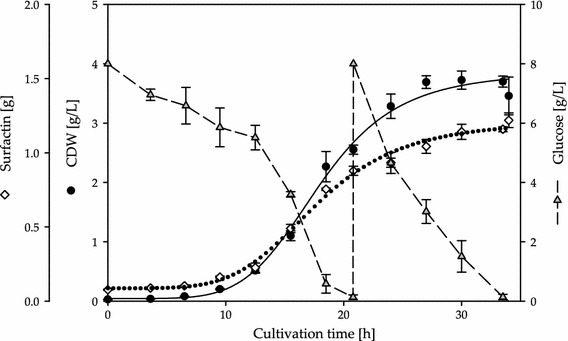
Time course of the fed-batch fermentation of *B. subtilis* DSM 10^T^ employing medium C. The time courses of CDW (*black dot*, [g/L]), Surfactin (*white rhombus*, [g]) and glucose (*grey triangle*, [g/L]) are displayed as mean values of two fermentations. Glucose was added after its complete consumption (23 mL of 450 g/L glucose, 20.83 h after inoculation). The *dotted* and *solid lines* represent logistic fits of CDW and mass of Surfactin

**Fig. 3:**
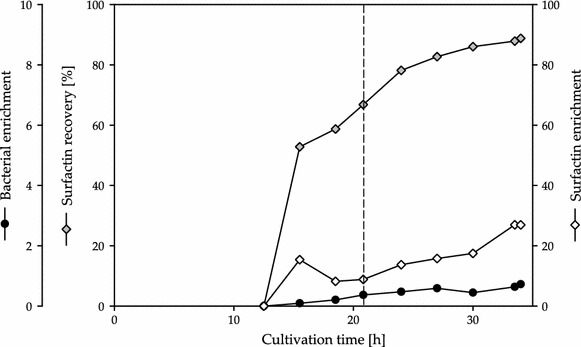
Time course of foam traps during fed-batch fermentation of *B. subtilis* DSM 10^T^ employing medium C. The values of bacterial enrichment (*black dots*), Surfactin recovery (*grey rhombus*) and Surfactin enrichment (*white rhombus*) are displayed as exemplary results of one fermentation. The addition of glucose is indicated by a *dashed line* after 20.83 h of cultivation

The bioreactor cultivations of Willenbacher et al. (2014) were performed as batch cultivations. As the optimized medium C is glucose limited batch cultivations would stop much earlier compared to cultivations with medium B. Therefore, a fed-batch cultivation was applied for the cultivation of *B. subtilis* DSM 10^T^ in the optimized medium C. The fermentation plot is shown in Fig. 2. Fermentations endured for 34 h and yielded a maximal CDW of 3.8 g/L. The decrease of glucose is visible until its complete depletion after 20.83 h of cultivation. Glucose was added to the culture broth to continue the cultivation (23 mL 450 g/L glucose), resulting in a glucose concentration of 8 g/L. The amount of glucose inside the bioreactor decreased again until its consumption after 34 h of cultivation. The increase of CDW and Surfactin followed a logistic growth behavior during cultivations, yielding 1.22 g Surfactin. The analysis of the foam traps is shown in Fig. 3 (example of one bioreactor cultivation). The Surfactin recovery increased during cultivations from 52 to 88 %, whereas Surfactin (15–27) and bacterial enrichment (0.1–0.7) remained nearly constant.

An overview about the pre-post comparison of the media B and C (Table 1) is given in Table 2. Various process parameters are listed in comparison to emphasize the effects of the different media. The growth behavior of *B. subtilis* DSM 10^T^ differed only slightly during employment of the optimized medium C. The value of Y_X/S_ decreased in comparison to the fermentation from Willenbacher et al. (2014) (Y_X/S_ = 0.20 g/g in contrast to Y_X/S_ = 0.27 g/g), but values for maximal growth rate µ and cultivation time remained on a similar level. However, the concentration of CDW increased significantly from 2.97 to 3.80 g/L during cultivation with medium C. The analysis of the foam traps identified a considerable decline in Surfactin enrichment, where maximal values of 101.92 decreased to 27.10. The Surfactin recovery decreased as well during employment of medium C in comparison to fermentations applying medium B (83.81 % instead of 91.96 %), although not as drastic as the Surfactin enrichment. In contrast, values for bacterial enrichment improved significantly with a mean value of 0.4 during application of medium C compared to the mean value during fermentations employing medium B (1.60). The production rate increased significantly as values for Y_P/X_ rose from 0.19 g/g (batch, medium B) to 0.26 g/g (fed-batch, medium C). Values for Y_P/S_ increased as well yielding 0.05 g/g instead of 0.03 g/g. The specific production rates q_Surfactin_ and vol. q_Surfactin_ achieved values of 0.009 g/(g h) and 0.022 g/(L h) [batch, medium B: q_Surfactin_ = 0.006 g/(g h), vol. q_Surfactin_ = 0.017 g/(L h)]. The improvement of the production rates becomes even more significant when analyzing the amount of produced Surfactin. The maximal Surfactin concentration in foam did not increase during cultivation employing medium C (3.67 g/L Surfactin in comparison to 3.99 g/L Surfactin applying medium B), but the total foam volume leaving the bioreactor increased from 334 mL (batch, medium B) to 435 mL (fed-batch, medium C). The collected amount of Surfactin inside the foam traps added up to 1.02 g Surfactin in fermentations employing medium C in contrast to 0.74 g Surfactin in fermentations applying medium B. In total 1.22 g Surfactin was produced employing the optimized medium C and a fed-batch strategy in comparison to 0.81 g Surfactin during batch fermentations applying medium B. This proves an enhancement of the Surfactin production of approximately 30 % based on the conversion of the fermentation strategy and medium optimization.

**Table 2:** Comparison of process parameters during fermentation of *Bacillus subtilis* DSM 10^T^ employing medium B and C

	Fed-batch	Batch
	The current study	Willenbacher et al. (2014)
Applied medium	C	B
Fermentation approach	Foam fractionation	Foam fractionation
Initial glucose conc.	8 g/L	40 g/L
Addition of glucose	23 mL of 450 g/L	–
Final glucose conc.	0 g/L	29.19 g/L
Cultivation time (h)	34	30
Max. CDW (g/L)	3.80	2.97
µ_max_ (h^−1^)	0.31	0.34
Max. c_Surfactin_ foam (g/L)	3.67	3.99
Foam volume (mL)	435	334
Surfactin in foam (g)	1.02	0.74
Overall Surfactin (g)	1.22	0.81
Y_P/X_ (g/g)	0.26	0.19
Y_X/S_ (g/g)	0.20	0.27
Y_P/S_ (g/g)	0.05	0.03
Int. q_Surfactin_ [g/(g h)]	0.009	0.006
Int. vol. q_Surfactin_ [g/(L h)]	0.022	0.017
Overall Surfactin recovery (%)	83.81	91.96
Max. Surfactin enrichment	27.10	101.92
Mean bacterial enrichment	0.41	1.60

The approach and results of *B. subtilis* DSM 10^T^ batch fermentation (Willenbacher et al. 2014) is compared to data collected during fed-batch fermentation of *B.* *subtilis* DSM 10^T^ employing the further optimized medium C

As a first conclusion it must be emphasized that Surfactin is not consistently produced throughout the cultivations. This has been taken into account during this study and it became possible to significantly enhance the Surfactin productivity for the strain *B. subtilis* DSM 10^T^. The substitution of the medium components NH_4_Cl and EDTA with (NH_4_)_2_SO_4_ and citrate, as well as the alteration of the glucose concentration (from 40 to 8 g/L) improved the production of Surfactin during shake flask experiments. Further shake flask cultivations revealed a general enhancement of Surfactin productivity independent from the employed *Bacillus* strains. The utilization of the improved medium would most likely also lead to better results for other *B. subtilis* strains. Comparable studies did not prefer low glucose concentrations, but failed to analyze concentrations below 10 g/L glucose. The comparison of fermentations employing the optimized medium plus a fed-batch strategy and fermentations applying the original medium in a batch process (Willenbacher et al. 2014) revealed an enhancement of Surfactin production of approximately 30 %.

## Additional file

Additional file 1. Shake flask cultivations analyzing different glucose concentrations and yield enhancement of other Surfactin producer strains.
