# Supplementary material for: Enhancement of Surfactin yield by improving the medium composition and fermentation process
Source: AMB Express. 2015 Aug 22;5:57. doi: 10.1186/s13568-015-0145-0 (PMC4546119; doi:10.1186/s13568-015-0145-0)
Supplement: Additional file 1. — Shake flask cultivations analyzing different glucose concentrations and yield enhancement of other Surfactin producer strains. [file 13568_2015_145_MOESM1_ESM.pdf]

Supplementary data for manuscript

"Enhancement of Surfactin yield by improving the medium composition and fermentation process"

Submitted to AMB Express on 2015-05-13

Judit Willenbacher[a,], Wladimir Yeremchuk[a], Teresa Mohr[a], Christoph Syldatk[a] and Rudolf Hausmann[b]*

[a]Institute of Process Engineering in Life Sciences, Section II: Technical Biology, Karlsruhe Institute of Technology (KIT), Engler-Bunte-Ring 1, 76131 Karlsruhe, Germany

[b]Institute of Food Science and Biotechnology (150), Section Bioprocess Engineering (150k), University of Hohenheim, Garbenstr. 25, 70599 Stuttgart, Germany

***Corresponding author:**	Judit Willenbacher
Address:	Engler-Bunte-Ring 1, 76131 Karlsruhe, Germany
Phone:	+49-721-608-46737
Fax:	+49-721-608-44881
Email:	judit.willenbacher@kit.edu

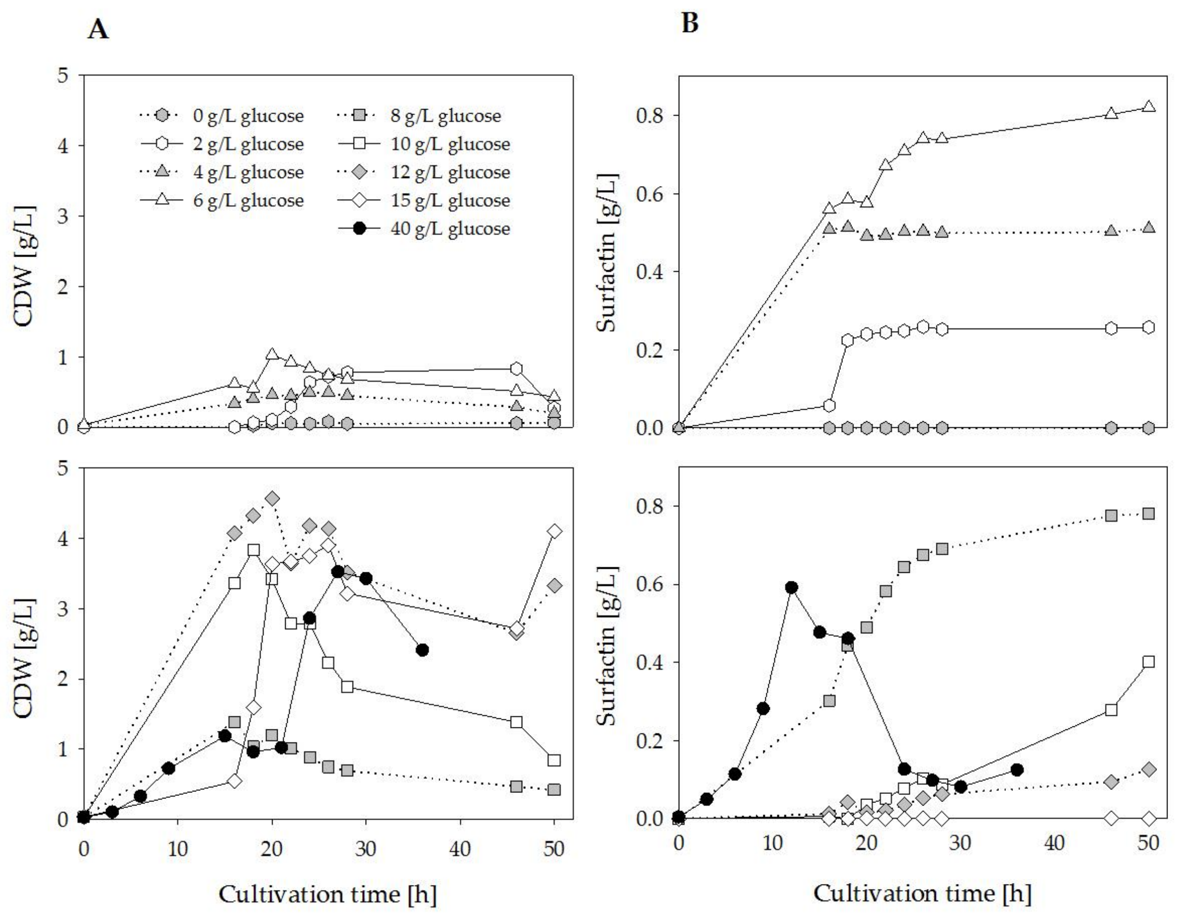

Figure S1 Time course of CDW and Surfactin concentrations of shake flask cultivations of *B. subtilis* DSM 10^{T} applying various glucose concentrations Cultivations in shake flasks were conducted employing 0 g/L (*grey hexagon*), 2 g/L (*white hexagon*), 4 g/L (*grey triangle*), 6 g/L (*white triangle*), 8 g/L (*grey square*), 10 g/L (*white square*), 12 g/L (*grey rhombus*) and 15 g/L (*white rhombus*) glucose. Data from earlier experiments employing the original 40 g/L are shown as *black dots*. The achieved CDW [g/L] is illustrated in **A**, the reached Surfactin concentration [g/L] is displayed in **B**. Shake flask experiments were conducted in duplicates and time-displaced flasks to illustrate a continuous course of CDW and Surfactin concentration

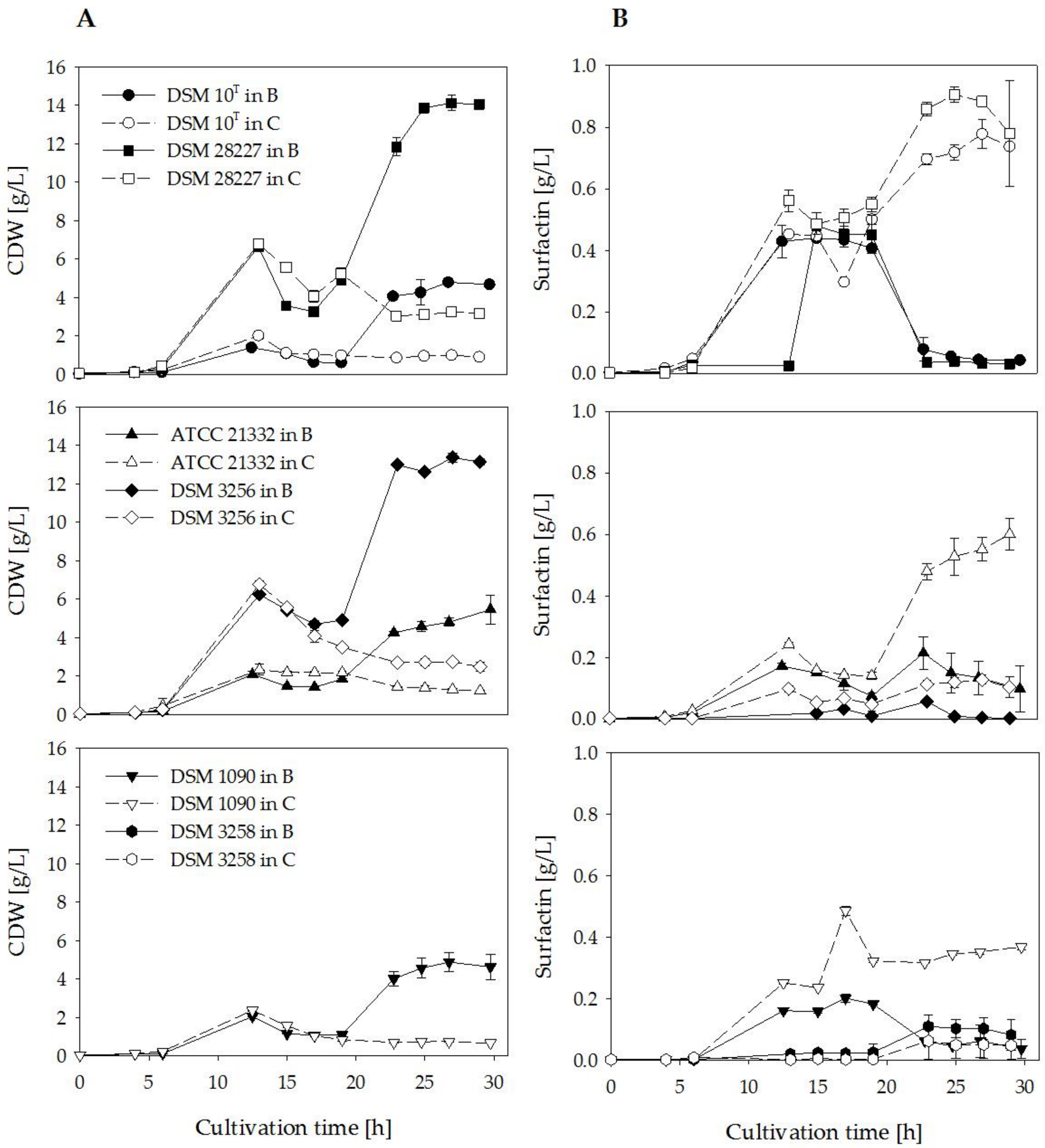

Figure S2 Time course of CDW and Surfactin concentration of various *Bacillus* strains cultivated in medium B and further optimized medium C In summary, six different Surfactin producers DSM 10^T (*dot*), DSM 28227 (*square*), ATCC 21332 (*regular triangle*), DSM 3256 (*rhombus*), DSM 1090 (*triangle upside down*) and DSM 3258 (*hexagon*) were cultivated in medium B (illustrated in *black*) and further optimized medium C (displayed in *white*). The results of achieved CDW [g/L] are shown in **A** (the pelleted growth of DSM 3258 did not allow a determination of the

OD_{600} absorption), whereas Surfactin concentrations [g/L] are illustrated in **B**. All experiments were conducted as duplicates and in time-displaced shake flasks to present continuous courses of CDW and Surfactin concentration
